# Optimizing preparation of renal biopsy specimens for TEM: a time-efficient approach to rapid diagnosis of kidney diseases

**DOI:** 10.1080/0886022X.2025.2522325

**Published:** 2025-06-26

**Authors:** Gen Wang, Xiaodong Zhu, Shaoshan Liang, Feng Xu, Dandan Liang, Caihong Zeng

**Affiliations:** National Clinical Research Center for Kidney Diseases, Jinling Hospital, Affiliated Hospital of Medical School, Nanjing University, Nanjing, China

**Keywords:** Renal biopsy, electron microscopy, specimen preparation, rapid diagnosis, kidney disease

## Abstract

Electron microscopic examination of renal biopsies is crucial for the diagnosis of kidney disease. In this study, we present an optimized protocol that significantly reduces processing time to 19.5 h, compared to 120 h with conventional methods, while preserving ultrastructural quality in 55 renal biopsy specimens. The modified method incorporates the use of higher concentrations of fixatives, shortened fixation duration, low-viscosity resin embedding, and the application of uncoated copper grids. Comparative analysis of renal specimens processed using conventional and optimized methods demonstrated equivalent preservation of critical ultrastructural features, including the glomerular basement membranes, podocytes and tubular epithelium. The total processing time was markedly reduced to 19.5 h, compared to several days with conventional methods. This rapid protocol enables timely ultrastructural evaluation of renal biopsies to facilitate prompt diagnosis and decision-making, highlighting the significance of optimizing diagnostic pathology methodologies for enhancing efficiency and maintaining accuracy.

## Introduction

Transmission electron microscopy (TEM), routinely used in most major nephrology centers, offers invaluable diagnostic insights into approximately half of native renal biopsies [1]. TEM’s high-resolution capabilities enable the identification of diverse immune and nonimmune substances within glomeruli, damage to epithelial or endothelial cells, intrinsic or acquired defects in glomerular basement membranes (GBM), and inclusion bodies within cells [2–4]. Accurate diagnosis of glomerular diseases, such as minimal change nephropathy, membranous nephropathy, lupus nephritis, membranoproliferative glomerulonephritis, early diabetic nephropathy, and thin basement membrane nephropathy, critically depends on ultrastructural findings [5]. This is particularly relevant in cases of isolated hematuria and hereditary nephritis, making TEM an indispensable tool for nephrologists and pathologists to examine patients with kidney disease [6].

The preparation of renal biopsy specimens for TEM involves several steps including fixation, dehydration, infiltration, embedding, ultrathin sectioning, and staining. Conventional procedures requires a specific duration and time-consuming [7]. Patients under nephrology care usually spend a duration of hospitalization ranging from 7 to 10 days, with a renal biopsy typically performed on the third day post-admission. Owing to the time-consuming TEM preparation procedures, coupled with the average turnaround time from the receipt of the sample in the laboratory to the final report was 26 days (interquartile range 6–64) [8], patients are generally unable to receive their TEM reports prior to hospital discharge. This is one of the significant reasons that hinders the widespread application of TEM in the diagnosis of kidney diseases. Alternative methods, such as high-pressure freezing and microwave-assisted processing, have been developed to accelerate sample preparation [9,10]. However, these techniques require specialized equipment, which limits their adoption in many clinical settings. Thus, optimizing conventional methodologies is crucial for increasing diagnostic efficiency in renal pathology laboratories. To address this challenge, the present study demonstrates an optimized method for preparing renal biopsy samples, which involves several modifications, including a higher glutaraldehyde concentration, shorter fixation and post-fixation times, the use of a low-viscosity resin, and a more efficient polymerization process. These measures collectively achieved a significant reduction in the overall sample preparation time while ensuring the preservation of kidney ultrastructures. Consequently, this study provides a rapid method for renal biopsy sample preparation, which effectively meet the clinical diagnostic needs for kidney diseases.

## Materials and methods

### Sample collection

In this study, 55 patients were selected from Jinling Hospital’s National Kidney Disease Clinical Medical Research Center between January and December 2023. These cases included various types of kidney diseases, including IgA nephropathy (*n* = 10), membranous nephropathy (MN) (*n* = 10), diabetic nephropathy (DN) (*n* = 10), focal segmental glomerulosclerosis (FSGS) (*n* = 10), lupus nephritis (LN) (*n* = 10), Alport syndrome (*n* = 2), light-chain deposition disease (LCDD) (*n* = 2) and Amyloidosis nephropathy (*n* = 1). All samples were obtained from diagnostic renal biopsy specimens that were immediately processed, with no exclusion due to inadequate tissue volume or fixation-related issues. The study has been reviewed and approved by the Jinling Hospital Ethics Committee(Reference number: 2025DZKY-024-01). Informed written consent was obtained from all individual participants included in the study.

### Sample processing

This study adopted a comparative methodology, which involved dividing the renal biopsy samples into two distinct groups: a conventional group and an optimized group.

In the conventional group, sample preparation followed the standard protocol for TEM imaging [11] (Table 1). Specifically, 1 mm^3^ blocks of renal biopsy specimens were rapidly prefixed 2.5% glutaraldehyde at 4 °C overnight. Subsequently, the tissue was rinsed in phosphate buffer and post-fixed within 1% osmium tetroxide for 2 h. The specimens were then subjected to a graded dehydration process using alcohol and acetone, followed by infiltration and embedding in Epon 812 resin. The resin was cured through a gradual temperature increase over a multi-day period: 12 h at 37 °C, 24 h at 45 °C, and 48 h at 60 °C. 80 nm ultrathin sections were cut and collected on formvar-coated copper grids, and contrasted with uranyl acetate and lead citrate prior to observation under the FEI Tecnai G2 Spirit transmission electron microscope.

**Table 1.** Comparison of the optimized method and conventional method procedures.

Procedures	Conventional method	Optimized method
Pre-fixation	2.5% glutaraldehyde overnight	3.75% glutaraldehyde for 2 h
Rinsing	wash in PBS for 15 min (repeat 3 times)	wash in PBS for 5 min (repeat 3 times)
Post-fixation	1% osmium tetroxide for 1 h	2% osmium tetroxide for 1 h
Rinse	deionized water for 15 min (repeat 2 times)	deionized water for 15 min (repeat 2 times)
	30% alcohol 30 min	50% alcohol 15 min
	50% alcohol 30 min	70% alcohol 15 min
Dehydration	70% alcohol 30 min	90% alcohol 15 min
	90% alcohol 30 min	absolute alcohol 15 min (repeat 3 times)
	absolute alcohol 30 min	acetone 15 min
	acetone 30 min (repeat 3times)	1:1 Eponate 12/acetone for 2 h
Infiltration	25% resin in acetone, and then 50%, 75%, three changes of 100% resin, all for 1 h, then 100% resin overnight	
Polymerization	12 h at 37 °C, 24 h at 45 °C, and 60 °C oven for 2 days	70 °C for 12h
Total duration	approximately 120 h	19.5 h

By optimizing the TEM preparation procedures for renal biopsy samples, the total time required from fixation to ready section was significantly reduced to 19.5 h.

In contrast, the optimized sample processing method involved several modifications (Table 1). During the pre-fixation stage, renal biopsy specimens were fixed in a higher concentration of 3.75% glutaraldehyde solution, with a reduced fixation time of 2 h. The postfixation step increased the concentration of OsO4 to 2%, and the fixing time was shortened to 1h to improve fixing efficiency. A low-viscosity Eponate 12 resin was employed in the infiltration and polymerization processes. This resin is notable for its short infiltration time and ability to achieve complete polymerization at 70 °C within 12-h duration. The staining and ultrastructure observation steps were consistent with those used in the conventional method.

### Ultrastructure observation

Extensive validation of the optimized method across a diverse range of glomerular diseases. This involved comprehensive comparative analyses between the optimized and conventional methods to determine the ultrastructural preservation of renal cells, including GBM, podocytes, mesangial cells and tubular epithelial cells, as well as electron-dense substances. The subcellular organelles of the cells were examined carefully. Detailed observations of various kidney diseases were performed to evaluate the reliability and diagnostic accuracy of the optimized method.

## Results

In contrast to the conventional method, which requires a considerably longer period of 120 h, the optimized method involves adjustments at several critical stages: prefixation, postfixation, washing, dehydration, infiltration, and embedding. Collectively these adjustments resulted in a marked reduction in the total preparation time to 19.5 h (Table 1). Notably, the fixation, infiltration, and embedding procedures experienced a considerable decrease in processing duration, from 105 h to 17 h, significantly expediting the overall process. This time-saving optimization is particularly valuable, as it allows for TEM ultrastructural examination as early as the day after the biopsy, thereby accelerating the diagnostic process and potentially improving patient care.

**Figure 1.**
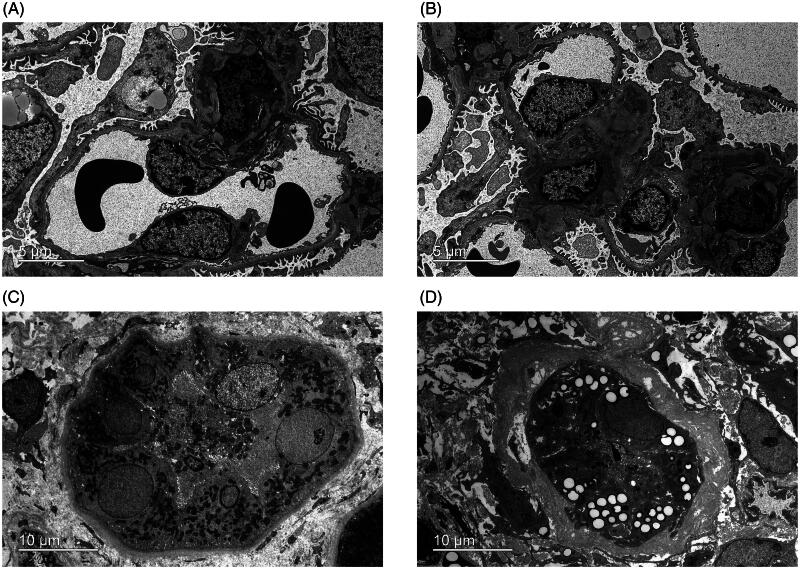
Ultrastructural analysis of IgA nephropathy reveals comparable ultrastructural components across conventional and optimized groups. (A–B) Examination of the conventional and optimized groups for IgA nephropathy both clearly reveal mesangial electron dense deposits, mesangial cells, podocytes, GBM and endothelial cells. (C–D) Similarly, both conventional and optimized groups exhibit clear ultrastructural characteristics of tubular cells and renal interstitium.

**Figure 2.**
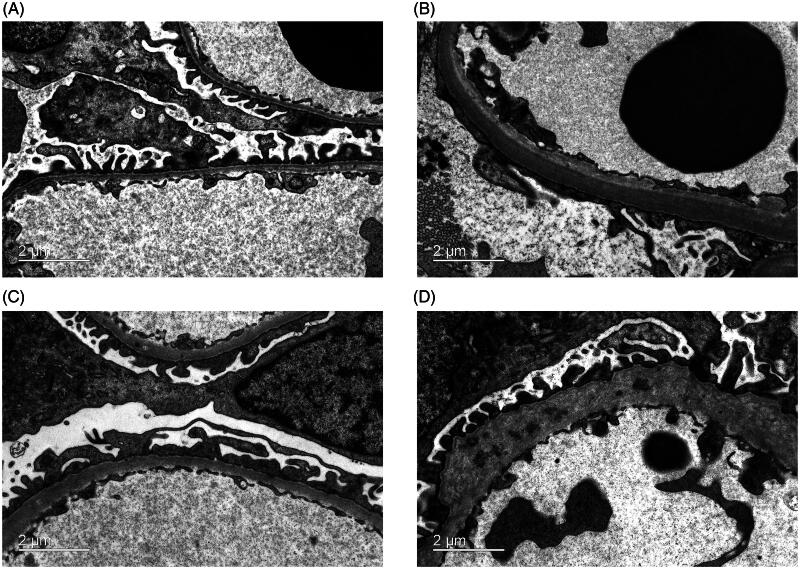
Ultrastructural preservation of the GBM and podocyte in the optimized preparation group. (A) Thin GBM was clearly observed in patients with TBMN. (B) DN exhibited GBM thickening with the absence of the middle dense layer. (C) Samples from patients with FSGS exhibited widespread fusion of podocyte foot processes. (D) Alport syndrome displayed characteristic features of GBM layering and loss.

**Figure 3.**
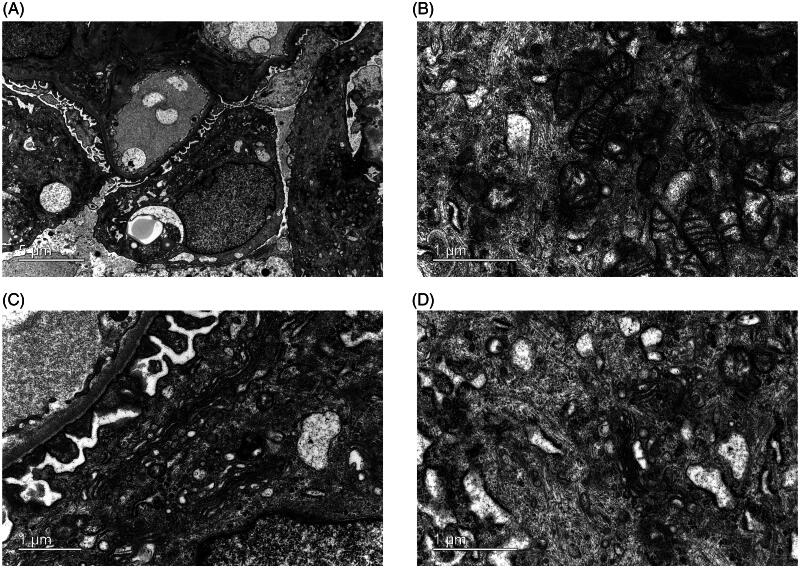
Ultrastructural examination of podocyte organelles using the optimized preparation method. (A) It provides a clear visualization of fine structures, including (B) mitochondria, (C) endoplasmic reticulum and Golgi apparatus, and (D) the cytoskeleton network. (B, C, and D are high magnifications of diagram A).

Ultrastructural preservation of renal biopsy specimens from patients with IgA nephropathy was examined using conventional and optimized methods. As shown in Figure 1(A), the conventional approach clearly displays characteristic mesangial electron-dense deposits and visualizes mesangial cells, podocytes, and endothelial cells. Similarly, the optimized method effectively preserved the key ultrastructural components of the mesangial electron-dense deposits, mesangial cells, podocytes, and endothelial cells (Figure 1(B)). Figure 1(C,D) clearly shows the well-preserved renal tubular cells and interstitium in both groups. GBM, a pivotal component of the glomerular filtration membrane, exhibits a variety of lesions in thickness, layering and even loss. These defects are associated with various of kidney diseases. Podocyte foot process effacement is characteristic of proteinuric glomerular disease. The optimized method ensures the good preservation and precise observation of these subtle ultrastructural alterations (Figure 2(A–D)). As illustrated in Figure 3(A), the ultrastructural examination of podocyte subcellular organelles in the optimized group was clearly identifiable. Specifically, the mitochondrial cristae (Figure 3(B)), endoplasmic reticulum and Golgi apparatus (Figure 3(C)), and cytoskeleton network (Figure 3(D)) exhibited characteristic structures with clarity.

**Figure 4.**
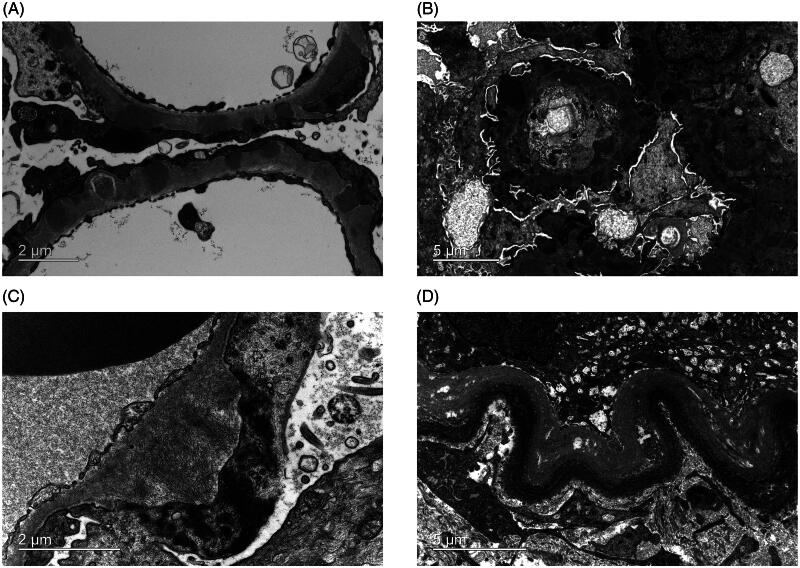
Ultrastructural features of various renal lesions using the optimized preparation method. (A-B) MN and LN immunocomplex electron-dense deposits, with absorption of dense deposits. (C) Amyloid fibrils in the intramembranous region. (D) LCDD powdery punctate electron-dense deposits distributed in the tubular basement membrane are clearly depicted.

The ultrastructural features of MN, LN, Amyloidosis, and LCDD were clearly visualized in the optimized preparation group. In MN, there is clear evidence of abundant subepithelial deposits and electron-lucent-like areas that are indicative of resorption of prior subepithelial immune complexes (Figure 4(A)). In diffuse proliferative lupus nephritis, the presence of immune complex deposits in the subepithelial and subendothelial regions, along with the mesangium, was clearly documented (Figure 4(B)). Branching amyloid fibrils of 8–12 nm in diameter were clearly oriented in the intramembranous region (Figure 4(C)). The definitive diagnosis of LCDD was facilitated by the clear detection of powdery punctate electron-dense deposits within the tubular basement membranes (Figure 4(D)). In conclusion, these findings confirm the optimized method’s high efficiency and quality of specimen preparation, supporting its application for ultrastructural analysis of renal biopsies.

## Discussion

Electron microscopy provides invaluable diagnostic insights into approximately half of native renal biopsies [1]. However, the conventional methods used for preparing renal tissue samples for TEM typically involve a relatively long sample processing time, often requiring several days or weeks [7]. This not only affects the timeliness of diagnosis, but may also have adverse effects on patient treatment. Addressing the need for an efficient protocol, with the aim of reducing the TEM sample preparation time, has emerged as a concern in renal pathology laboratories. Several rapid alternatives, such as microwave-assisted processing and high-pressure freezing (HPF), have emerged [9,10]. Microwave techniques utilize electromagnetic radiation to accelerate chemical reactions, reducing fixation, dehydration, and resin polymerization times to as little as 4–8 h. Similarly, HPF immobilizes cellular structures within milliseconds, preserving ultrastructure with minimal artifacts, followed by freeze-substitution over 24–48 h. However, these methods necessitate specialized equipment and may not be feasible for routine use in resource-limited laboratories. The optimized protocol significantly enhances efficiency, reducing processing time from 120 h to 19.5 h—a 6-fold decrease in technician time. While material costs (e.g., glutaraldehyde, resin) remain comparable, the elimination of specialized equipment (e.g., microwave processors, high-pressure freezers) enhances the practical applicability and makes this method cost-effective for resource-limited laboratories. Nevertheless, our method strikes a pragmatic balance between speed, cost, and diagnostic accuracy, addressing a critical need for rapid TEM integration into clinical workflows.

Almost every TEM laboratory employs unique variations in methodologies, including differences in fixation, post-fixation processes, dehydration techniques, embedding procedures, and preparation of semi-thin and thin sections [12]. The most common fixation strategy is double fixation with glutaraldehyde and osmium tetroxide. The appropriate optimization of fixation parameters, including the concentration of the fixative, duration of fixation, sample size, and temperature of the fixative, leads to optimal preservation of the ultrastructure. In the conventional method, low 1–2% concentrations of glutaraldehyde are typically used as a fixative, which to some extent affects the fixation efficacy of the tissue. In the optimized procedure, the glutaraldehyde concentration was increased to 3.75% for 2 h, which enhanced fixation efficacy and shortened fixation duration. Osmium tetroxide functions as an oxidizing agent that enables the binding of lipids, particularly those found within the cell membrane [13]. In this study, the concentration of osmium tetroxide was increased to 2% to improve the preservation of the ultrastructure, whereas the fixed time remained unchanged. Notably, extended exposure to osmium tetroxide can lead to protein degradation, which subsequently renders the biological material brittle.

The Eponate 12 embedding resin takes advantage of its low viscosity, diffuses more rapidly into tissues and requires a shorter infiltration time. In conventional methods, infiltration involves the gradual exchange of increasing concentrations of resin. However, we have shown that the quality of the quick infiltration process is as good as that of conventional procedures, which take many days to complete. After changing to pure Eponate 12 resin, the samples are ready for polymerization, which was accomplished within 16–24 h at 60 °C. An increase in the temperature facilitates rapid polymerization. In practice, we usually leave samples in an oven at 70 °C overnight to polymerize and find that they work quite well. In fact, some previous studies have shown that polymerization at 100 °C has also yield acceptable results [14]. In addition, the optimized method employed uncoated copper grids. This facilitates ultrathin sectioning, which freely interacts with the staining solutions on both surfaces. This avoids interference from formvar or carbon films, which significantly improves the contrast and clarity of the images. However, there exists a potential limitation of this approach that could adversely affect section stability during handling or staining procedures.

In conclusion, this optimized protocol for renal biopsy specimen preparation for TEM significantly reduces processing time while maintaining ultrastructural integrity, making it a cost-effective and efficient alternative to conventional methods. The method’s core principles, such as using higher fixative concentrations, shorter fixation times, and low-viscosity resins, suggest it could be adapted for other tissue types beyond renal biopsies. However, this would require tissue-specific validation to ensure ultrastructural preservation. Further multi - center validation and testing across a broader range of kidney diseases and tissue types would strengthen the protocol’s applicability. This could ultimately support more rapid, accurate diagnosis and management of kidney diseases and other conditions requiring ultrastructural analysis.
